# Koch and the Consumption Cure

**Published:** 1891-01-03

**Authors:** 


### KOCH AND THE CONSUMPTION CURE.

The event of 1890 which caused the greatest excitement in the medical world, and perhaps in the world generally, was the announcement that Dr. Robert Koch, the well-known Professor of Hygiene at Berlin, had discovered a cure for consumption. The news flew abroad over the civilised world like the annuncement of a great victory, or the death of the monarch of one of the leading states. In every country, in every village, in almost every family, there was a momentary illumination of the dark chambers of sorrow, and the quickening of hopes that were well nigh dead. All the world has looked on whilst the sick and the dying in melancholy troops have hurried from the remotest corners of civilization to the scientific magician of Berlin. But the promise was too great: human intelligence and human skill have not yet learnt to exorcise by a word or an inoculation the hateful spirit of consumption. This much at least is unhappily all too clear. That Koch has discovered or manufactured a substance of great potency nobody will deny; that eminent men of the highest scientific attainments, like Sir Joseph Lister and others, have been ready to give to Koch a loyal confidence and an almost unquestioning faith, is as evident to the world as it is honourable to themselves; that lupus and some other forms of tubercular disease are powerfully, and, for the time, favourably influenced by Koch's treatment is undeniable. But that a remedy has been discovered for tubercular consumption which is worthy of being called by the simple but definite name of a "cure," is plainly not the case. There has been up to the present time not even an attempt made to formulate the facts for and against Koch's discovery; to set them out in scientific array, and to elaborate an impartial judgment thereupon. As yet everything has been excitement, confusion, and haste. Names have been weighed instead of facts, and reputations put in place of arguments. This is not science—it never can be science. Plato may be every scientific man's friend, but truth is his divinity. We are all loyal to truth in the long run; not only Englishmen but Americans, not only Americans but Frenchmen, not only Frenchmen but Germans, not only Germans but the world. If Koch's discovery should ultimately prove to be a cure for consumption, this country and all countries, and most of all the adverse critics of all countries, will stand uncovered in the presence of the great discover and acknowledge him Primus and Prince. In the meantime science keeps her eyes open and waits.

